# Management of triggering factor effects in sensitive skin syndrome with a dermo‐cosmetic product

**DOI:** 10.1111/jocd.16529

**Published:** 2024-09-18

**Authors:** Helena Polena, Arnaud Fontbonne, Elise Abric, Guillaume Lecerf, Marlène Chavagnac‐Bonneville, Alain Moga, Nathalie Ardiet, Sandra Trompezinski, Michèle Sayag

**Affiliations:** ^1^ Research and Development Department NAOS Ecobiology Company Aix‐en‐Provence France; ^2^ NAOS Institute of Life Science Aix‐en‐Provence France; ^3^ QIMA Synelvia Labège France

**Keywords:** biomarkers, ecobiology, sensitive skin, skin barrier, skin physiology

## Abstract

**Objective:**

Environmental factors are important in the generation or aggravation of sensitive skin syndrome (SSS). Creams can be useful for patients with SSS, particularly when triggering factors cannot be avoided. Several clinical studies have investigated the safety and efficacy of specific creams in patients with SSS, but the majority were assessed with a single type of triggering factor and were non‐comparative. Therefore, this study's aim was to investigate, the benefit of a specific dermo‐cosmetic product in response to physical and chemical factors in subjects with SSS.

**Methods:**

Three clinical studies were performed on subjects presenting SSS. The physical impact was assessed in a stripping test, and in a randomized intra‐individual study with a newly developed heat–cold stress model. To assess chemical factors, a capsaicin test on the nasolabial fold was performed.

**Results:**

The product significantly reduced the increase in skin microcirculation caused by stripping after 30 min versus. The untreated condition (45.8% vs. 62.0%; *p* < 0.01). Immediately and at D28, the product induced a significant increase in skin hydration even after a heat–cold stress, while the overall score of unpleasant symptoms significantly decreased compared with the control (8.1 vs. 10.7 and 3.7 vs. 8.0, respectively; *p* < 0.01). Regarding chemical factors, a significant difference in the sensation intensity (*p* < 0.001) was observed after capsaicin stress, also in terms of the sensation duration due to the product application versus the control (192 s vs. 403 s; *p* < 0.001).

**Conclusion:**

These studies show that topical application of a dermo‐cosmetic product can prevent unpleasant symptoms and improve the skin state in SSS exposed to physical and chemical triggering factors.

## 1 INTRODUCTION

Sensitive skin syndrome (SSS) is a common skin condition reported to occur in over 50% of the world's population.^1^ It is defined by the occurrence of unpleasant sensory sensations, such as stinging, burning, pain, pruritus, and tingling, in response to physical, chemical, or psychological factors that normally do not provoke such sensations.^2^ Sensitive skin can have a normal appearance or be accompanied by erythema, on the face and/or other body areas. While further studies are needed to better understand the pathophysiology of SSS, recent studies of SSS have shown that neurogenic inflammation is a key feature and is frequently associated with skin barrier abnormalities.^1^

Environmental and psychological factors play important roles in the generation or aggravation of SSS. Consequently, once it has been diagnosed, identification of the triggering factors is mandatory to be able to avoid them as much as possible. SSS triggering factors can be physical (mechanical, cold, heat, UV, or wind), chemical (cosmetic, water, or pollutants), and occasionally psychological (stress). Furthermore, SSS can also be exacerbated by an allergic setting.^1^ The unpleasant sensory sensations occur within 1 h following the exposure and can persist for minutes or even hours.^3^ Cosmetic products appear to be the main triggering factors, as reported by Misery et al., due to the potential irritant ingredients in their composition.^4^ According to this review, following cosmetics, the most frequent triggering factors are wet air, air conditioning, temperature variation, heat, water, pollution, dry air, cold, wind, sun, and emotion.

Nevertheless, since dry skin can be associated with SSS symptoms, specifically tailored cosmetic products can be useful for patients suffering from this syndrome, particularly when the triggering factors, such as heat, cold, and/or pollution, cannot be avoided. Topical creams can restore skin barrier function as well as protect and strengthen the skin barrier exposed to environmental stress.^1^, ^5^ Furthermore, patients with SSS require products that improve skin sensitivity.^3^

Several clinical studies have investigated the safety and efficacy of specific topical products in patients with SSS, and they have demonstrated improvement of the skin barrier^6^, ^7^ and the clinical signs in response to chemical stress^6^, ^7^, ^8^, ^9^, ^10^, ^11^ or physical stress.^11^, ^12^, ^13^ However, to our knowledge, only one study to date has investigated both chemical and physical stresses as well as included a vehicle‐control group.^11^ The aim of this study was to investigate the safety and efficacy of a specific dermo‐cosmetic product (Laboratoire Bioderma, NAOS Ecobiology Company, France) in response to several types of triggering factors (mechanical, heat–cold, irritant, and pollution) in subjects with SSS. This cream contains active ingredients that inhibit the neurogenic inflammation and that restore the skin barrier, thereby counteracting the triggering factor to provide immediate relief of sensitive skin and prevention of long‐term consequences. This novel ecobiological approach considers the skin, in relation to its environment, as an ever‐evolving ecosystem, for which the natural resources and mechanisms must be preserved,^14^, ^15^ and can be applied to develop specifically tailored skincare products.^16^

## 2 MATERIALS AND METHODS

### 2.1 Product and active complex

The studied product is an emulsion formulated for sensitive skin (Laboratoire Bioderma, NAOS Ecobiology Company, Aix‐en‐Provence) containing an active complex of four active ingredients, which is also formulated in a vehicle for the in vitro preclinical study on a UV‐pollution (see composition in Table 1).

**TABLE 1:** Composition of the different products and complex used in the studies.

Name	Composition
Studied product	Aqua/water, glycerin, dicaprylyl ether, propylheptyl caprylate, butyl glycol, glyceryl stearate citrate, glycol palmitate, squalane, sucrose stearate, *Mangifera indica* (Mango) seed butter, pentyl glycol, acrylates/C10–30 alkyl, acrylate crosspolymer, caprylyl glycol, carnosine, hydroxyethyl acrylate/sodium acryloyldimethyl taurate copolymer, mannitol, xylitol, tocopherol, *Salvia miltiorrhiza* flower/leaf/root extract, rhamnose, glycine soja (soybean) oil, polysorbate 60, sorbitan isostearate, and palmitoyl tetrapeptide‐10
Complex of active ingredients	*Salvia miltiorrhiza flower/leaf/root* extract, palmitoyl tetrapeptide‐10, carnosine, and tocopherol
Vehicle	Aqua, sodium polyacrylate, glycerin, hydrogenated polydecene, pentylene glycol, 1,2‐hexanediol, caprylyl glycol, polysorbate 20, and sodium citrate
Neutral cream	Aqua, caprylic/capric Triglyceride, glyceryl stearate citrate, sucrose stearate, xanthan gum, pentylene glycol, sodium polyacrylate, 1,2‐hexanediol, caprylyl glycol, citric acid, and glycerin

For the clinical study under heat–cold stress conditions, a neutral cream was used as a control of a standard cream (see composition in Table 1).

### 2.2 Ethics and exclusion criteria for in vivo studies

All in vivo studies complied with the Declaration of Helsinki, Good Clinical Practice Guidelines, and local laws and regulations. No approval from local ethics committees was required according to the local regulatory guidelines. All the subjects provided written informed consent prior to their participation in the study. Only women were recruited because they have less hair on the forearm and are more representative in the panel of SSS.^17^ The standard exclusion criteria were as follows: pregnant or breastfeeding women or women who intended to become pregnant, cutaneous pathology of the studied skin area, known allergy to certain cosmetic or dermato‐pharmaceutical products, the use of topical or systemic treatment in prior weeks that could potentially interfere with the assessment, undergone surgery under general anesthesia or excessive exposure to sunlight in the month preceding the study, and subjects enrolled in another clinical study during the study period of the studied areas.

### 2.3 Clinical study under mechanical stress (stripping test) conditions

In an open intra‐individual study, 22 women with phototype I to III (according to the Fitzpatrick scale) from 18 to 60 years of age were included. The study was carried out in a clinical center in Poland (Eurofins, Dermscan Poland). The additional exclusion criteria to the standard criteria previously described were as follows: hair, beauty spot or problems with the circulation of the studied area, and the use of topical or systemic treatment in prior weeks with the potential to interfere with the assessment of the cutaneous acceptability of the study product including treatments acting on the cutaneous microcirculation.

After 30 min of acclimation to an airconditioned environment (22 ± 2°C, relative humidity 35%–55%), mechanical erythema was induced by five to ten strippings using silver tape patches on two areas on the forearms before the product application: one untreated and one treated with the product applied at 2 μL/cm^2^ by a technician. Image acquisitions of the cutaneous microcirculation were performed using a Tivi700® device before (t0), immediately (ti), and 30 min (t30) after the strippings. The results are expressed as the average of the percentages of the stripping‐induced increase in microcirculation (Δ%) calculated for each area according to the following formula:

$$\Delta \%=\left(\left(V_{\text{34𝐭30}}-V_{\text{34𝐭34𝐭𝟎}}\right)/\left(V_{\text{34𝐭i}}–V_{\text{t0}}\right)\right)\times 100,$$

with *V* being the value obtained.

### 2.4 Randomized clinical study under heat–cold stress conditions

#### 2.4.1 Subjects and study design

In a randomized intra‐individual (split face) clinical study under dermatological control, 30 women with phototype I to III and 25–50 years of age with sensitive skin and living in a polluted environment (city or nearby) were included. All subjects declared to have a sensitive skin and a Burden of Sensitive Skin (BoSS) questionnaire score ≥20 on the day of inclusion.^18^ All also answered positively to questions #5 and #6 of the BoSS questionnaire about impact of climatization and urban pollution respectively.^18^ The study was carried out in a clinical center in France (CPP Initiatives/Dermatec, France). The additional exclusion criteria to the standard criteria previously described were as follows: irritation, hair, scar, tattoo, spot on the studied skin area, wearing a pacemaker (which is not compatible with the device used for heat–cold stress), and recent exposure to sunlight and/or having used self‐tanner on the face and/or tanning dietary supplement in the 4 weeks preceding the study.

Twice daily (morning and evening), from Day 0 (D0) to D27, the subjects applied the studied product on half of their face and neck, and a neutral cream as control on the other half according to randomization, after skin cleansing with the subjects' usual hygiene product. Since the study occurred during summer, a sunscreen (Photoderm MAX Crème SPF50+, Laboratoire Bioderma, NAOS Ecobiology Company, France) was applied on all of the face and neck once a day (morning).

#### 2.4.2 Heat–cold stress

The heat–cold stress involved three successive heat–cold cycles applied on the subject's half‐face, monitored by a thermal camera. The heat stress was performed with a radiofrequency system to reach a surface temperature of 45 ± 5°C. The cold stress was performed immediately afterward with a cold system blowing dry air pulsed to reach a cutaneous surface temperature of 15 ± 5°C.

#### 2.4.3 Efficacy and safety assessment

At D0 and D28, the investigator assessed the cutaneous hydration using a Corneometer®, the cutaneous skin barrier function by measuring the trans‐epidermal water loss (TEWL) using a Vapometer® as well as the intensity of the redness of each subject's half‐face using an 11‐point scale (0 = none, 1–3 = slight intensity, 4–6 = moderate intensity, 7–9 = high intensity, 10 = very high intensity). Using the same 11‐point scale, the subjects evaluated the unpleasant sensations associated with sensitive skin: prickling, heating, burning, and tightness at D0 and D28. The overall scoring of unpleasant symptoms associated with SSS was assessed by summing the score of each separate parameter (redness, prickling, heating, burning, and tightness).

At D0, after a single product application and after heat–cold stress followed by another product application, a standardized questionnaire with a 4‐point scale (agree, somewhat agree, somewhat disagree, and disagree) was used to evaluate the skin hydrating, protecting, soothing, and comfort effects. At the final visit, the overall efficacy of the study product was assessed by the subjects using the same scale. The answers “agree” and “somewhat agree” were both considered to be positive answers.

#### 2.4.4 Safety assessment

The safety assessment was based on the occurrence of adverse events from D0 to D28 reported by the subjects in the daily log, and clinical and functional signs between D0, before and after product application, and D28 on a four‐point scale. The evaluation at D28 by the dermatologist of the overall cutaneous acceptability was assessed using a four‐point rating scale (very good, good, moderate, and bad).

#### 2.4.5 Biomarker analysis

At D0 and D28, before and after heat‐cold stress time points, non‐invasive samplings were performed with a swab sampling kit supplied by QIMA Synelvia. The sampling was repeated twice per area and stored at −20°C. IL‐1α and calprotectin S100A8/9 were extracted from the supernatants and measured using a specific ELISA kit according to the supplier's instructions (R&D Systems). These assays were performed by ELISA in “sandwich” mode with a monoclonal antibody specific for IL‐1α or calprotectin S100A8/9.

The raw data were analyzed using Microsoft Excel® software and GraphPad PRISM® software R version 4.1.0 (2021‐05‐18).

### 2.5 Clinical study under chemical stress (capsaicin test) conditions

The study was carried out in a clinical center in Poland (Eurofins, Dermscan Poland) according to a protocol previously describes^19^ with C1 = 3.16 × 10^−5^% of capsaicin, C2 = 1.10 × 10^−4^%, C3 = 3.16 × 10^−4^%, C4 = 1.10^−3^%, and C5 = 3.16^−3^%. Twenty‐two women were recruited with phototype I–IV and from 18 to 60 years of age with sensitive and reactive face skin (had to be reactive to capsaicin concentrations of C1, C2, or C3 on the pre‐inclusion day). In addition, subjects did not wear make‐up on the day of the visit. Briefly on D0, after cleansing a capsaicin solution was applied to the nasolabial folds (n + 1 concentration according to the detection threshold defined at the pre‐inclusion). During the minute following the capsaicin application, the subjects were asked regarding the sensations felt (stinging, burning, itching, or other) and intensity using a six‐point scale (from 0 = no sensation to 5 = painful) on both nasolabial folds. Then after the product application to a randomly defined nasolabial fold, the subjects were asked regarding the sensations felt on both nasolabial folds immediately (30 s) and at 3, 6, and 9 min after product application. Afterwards, the subjects continued to apply the product to their face twice daily (morning and evening) for 28 days. Same as previously done on the pre‐inclusion day, on D28, a capsaicin test was performed.

The results are expressed as mean scores for the intensity sensation and as duration means for the sensation duration (in seconds) at D0, and as the reactive thresholds to capsaicin at pre‐inclusion and D28 calculated as the mean of the concentration number.

### 2.6 Statistical analysis

For the stripping test, statistical analysis was performed using the paired Student's *t*‐test.

For the capsaicin test and randomized clinical study under heat–cold stress conditions, if normality was proven by the Shapiro–Wilk test, the Tukey test was used for the cutaneous hydration rate and TEWL analyses, and the Wilcoxon signed‐rank test for the overall score of sensitive skin symptoms and a Student's *t*‐test was used, for the capsaicin test and biomarker analysis otherwise the non‐parametric Wilcoxon signed‐rank test was used. For all studies, *p* < 0.05 values were considered to indicate statistical significance (*p < 0.05, **p < 0.01, and ***p < 0.001).

## 3 RESULTS

### 3.1 Assessment under physical stress conditions: stripping test

**FIGURE 1:**
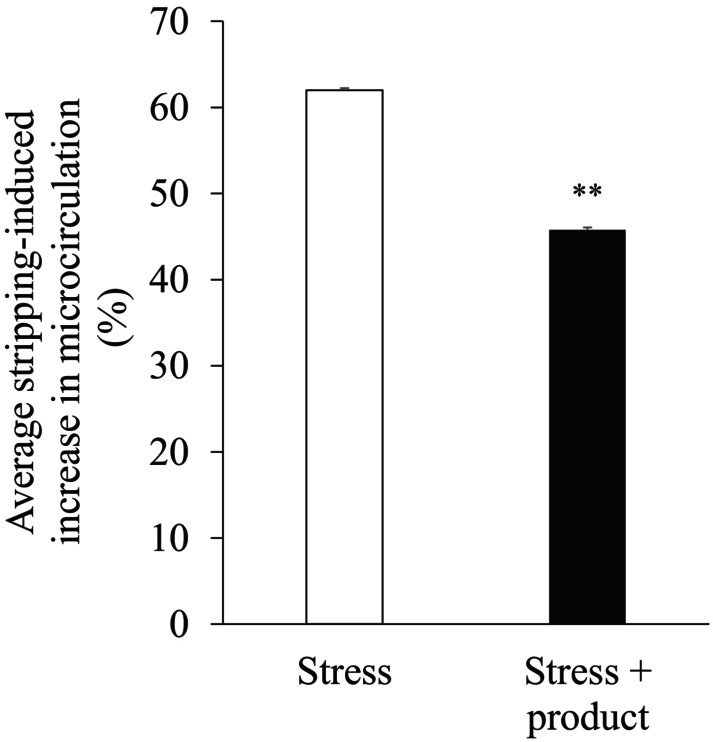
Efficacy of the product under mechanical stress conditions induced by stripping via assessment of the microcirculation 30 min after the stress (as a percentage). **p < 0.01.

To evaluate the immediate soothing effect of the product after mechanical stress, 18 women with a mean age of 42 ± 2 years (21 to 55) had their microcirculation analyzed 30 min after stripping (four presented aberrant values). The product significantly reduced (by 16.2%) the microcirculation compared with the untreated condition (45.8% vs. 62.0%; *p* < 0.01; Figure 1). No adverse reactions were observed during the study.

### 3.2 Assessment under physical stress conditions: heat–cold cycles

**FIGURE 2:**
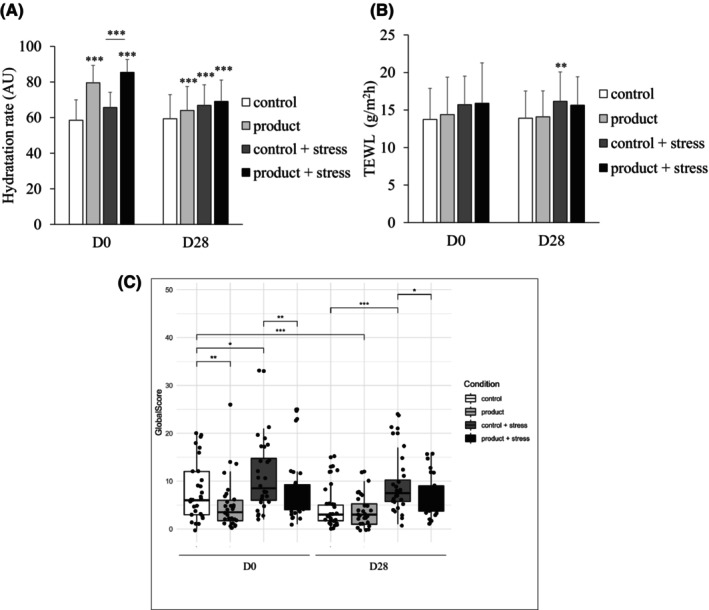
Efficacy of the product before and after heat–cold stress via assessment of the hydration rate, (A) TEWL, (B) overall score of unpleasant symptoms of SSS (C) at D0 and after 28 days of product application. Statistical analyses were performed versus control conditions: *p < 0.05; **p < 0.01; and ***p < 0.001.

The heat–cold stress, a major triggering factor of physical stress for sensitive skin, was assessed via a new in vivo protocol after just one application of the product and after 28 days of application in 30 women with a mean age of 37.9 years, as described in Table 2. Immediately after the application, the product induced a significant increase in the cutaneous hydration rate of the epidermis superficial layers of 45% compared with the control (79.6 vs. 58.5, respectively; *p* < 0.001) (Figure 2A) and even higher (55%) after a heat–cold stress (85.4 vs. 58.5, respectively; *p* < 0.001). Similarly, at D28, the product induced a significant increase in the cutaneous hydration rate of 15% compared with the control (64.0 vs. 59.3, respectively; *p* < 0.001), and 24% after another heat–cold stress (69.1 vs. 59.3, respectively; *p* < 0.001). In this model, at D28, the heat–cold stress also induced a significant increase in the hydration rate compared with the control (66.8 vs. 59.3, respectively; *p* < 0.001). For the TEWL, no significant increase was observed in heat–cold stress with the product condition compared with the control at D28, unlike with the heat–cold stress condition alone (Figure 2B), suggesting that the product prevented an increase in the TEWL after 28 days.

**TABLE 2:** Description of the studied population of the clinical study under heat–cold stress conditions. SD, standard deviation; BoSS, Burden of sensitive skin.

	*N* = 30
Age (years)	
Mean (±SD)	37.9 (±)
Min	24
Max	51
Gender	
Female	100%
Population type	
Caucasian	100%
Skin type	
Dry	40%
Mixed	53%
Normal	7%
Phototype	
I, II	43%
III	57%
Smoker	
Yes	30%
No	70%
Polluted environment	
Living in a city or nearby	100%
Sensitive facial skin	
BoSS ≥20, whereas questions regarding the climatization and urban pollution ≥2	100% 77%

**FIGURE 3:**
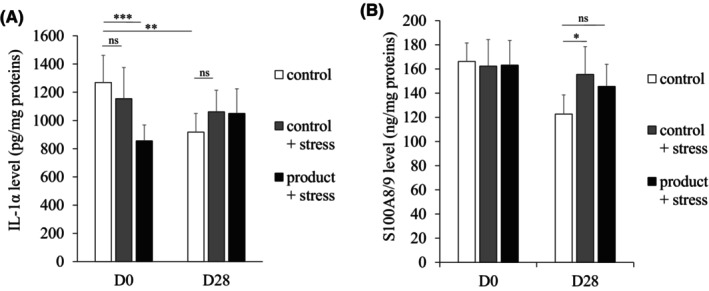
In vivo biomarker assessment in sensitive skin under heat–cold stress conditions after 28 days of product application: IL‐1α (A) and S100A8/9 (B). *p < 0.05; **p < 0.01; and ***p < 0.001.

Immediately after the application, the product induced a significant decrease in the overall score of the unpleasant symptoms associated with SSS of 39% compared with the control condition at D0 (7.7 vs. 8.0, respectively; *p* < 0.01), but not at D28 (Figure 2C). The heat–cold stress induced a significant increase in the overall score compared with the control condition at D0 (10.7 vs. 8.0, respectively; *p* < 0.05) and at D28 (8.8 vs. 4.3, respectively; *p* < 0.001). Interestingly, the overall score after the heat–cold stress condition followed by product application was significantly lower (by 29%) compared with that of the stress condition alone at D0 (8.1 vs. 10.7, respectively; *p* < 0.01). After 28 days of application, the product induced a significant decrease in the overall score of 50% compared with the control condition at D0 (3.7 vs. 8.0, respectively; *p* < 0.001) and of 21% after stress compared to stress condition alone at D28 (7.8 vs. 8.8, respectively; *p* < 0.05) (Figure 2C). After the application of the product, the subjects found that their skin was moisturized, protected against external stresses, soothed, and comfortable at D0 and after the heat–cold stress at D0 and D28 (Table 3). Over 83% of the subjects were very satisfied with the product's efficacy at D28. Concerning the IL‐1α levels, no significant effect of the heat–cold stress was observed compared with the control condition, both at D0 and D28, but after 28 days of product application corresponding to the control condition, the IL‐1α protein level was significantly reduced from D0 to D28 (1269.0 to 917.6 pg/mg; *p* < 0.01) (Figure 3A). The heat–cold stress associated with the product application induced a significant decrease of 32.6% in the IL‐1α protein level at D0 compared with the control (1269.0 vs. 855.4 pg/mg, respectively; *p* < 0.001). At D28, the S100A8/9 protein level was significantly increased by the heat–cold stress compared with the control condition (155.5 vs. 122.7 ng/mg, respectively; *p* < 0.05), but not under the stress condition with the product (145.6 vs. 122.7 ng/mg, respectively) (Figure 3B), suggesting that the product protected from the increase induced by stress. The product was well tolerated by all subjects.

**TABLE 3:** Subjective efficacy of the product under heat–cold stress conditions according to the subjects at D0 before and after stress and on D28 after stress (as a percentage).

	D0		D28
	Product	Product + stress	Product + stress
Your skin is moisturized	93	100	97
Your skin feels protected	87	93	97
Your skin is soothed	83	90	97
Your skin is comfortable	93	87	97

### 3.3 Assessment under chemical stress conditions: capsaicin test

**FIGURE 4:**
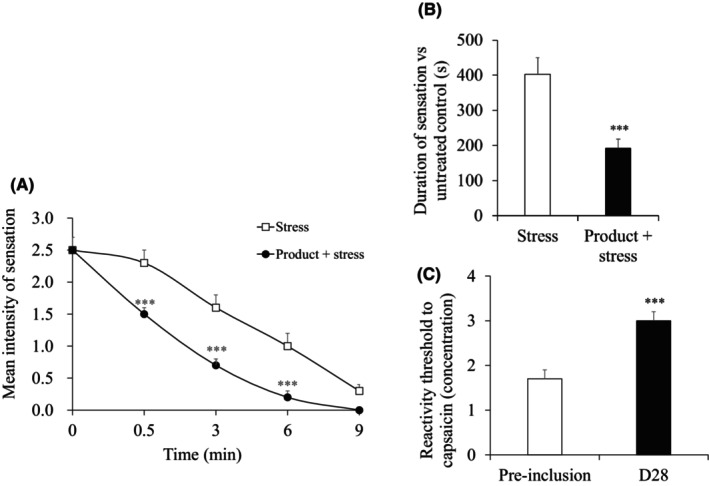
Efficacy of the product under chemical stress conditions induced by capsaicin via assessment of the sensation intensity for 9 min, (A) the sensation duration after stress at D0 (B) and the reactivity threshold after 28 days of product application versus the pre‐inclusion day in concentration (C1 = 3.16 × 10^−5^%, C2 = 1.10 × 10^−4^%, or C3 = 3.16 × 10^−4^%) (C). ***p < 0.001.

For 22 women with sensitive and reactive skin on the face (mean age 48 ± 2 years, 18–60), the product application significantly decreased the intensity of sensations induced by capsaicin stress (*p* < 0.001) by 43% after 30 s (1.5 vs. 2.5, respectively), by 73% after 3 min (0.7 vs. 2.5, respectively), by 91% after 6 min (0.2 vs. 2.5, respectively), and by 100% after 9 min (Figure 4A). Compared with the stress area, a significant difference (*p* < 0.001) was observed after 30 s (1.5 vs. 2.3, respectively), 3 min (0.7 vs. 1.6, respectively), and 6 min (0.2 vs. 1.0, respectively). At D0, the duration of the sensation after capsaicin stress was also significantly reduced by the product application (192 vs. 403 s, respectively; *p* < 0.001) (Figure 4B). After 28 days of product application, a significant increase of 78% in the minimal concentration of capsaicin detected by the subjects compared with the one determined at the pre‐inclusion (3.0 vs. 1.7, respectively; *p* < 0.001; Figure 4C). This effect was observed in 90% of the subjects. No adverse reactions were observed during the study.

## 4 DISCUSSION

The three clinical studies demonstrated first that the dermo‐cosmetic product significantly reduced or prevented the impact of several triggering factors: stripping, heat–cold stress, and capsaicin reactivity immediately and after 28 days of application. Second, without stress, after a single application, the product increased skin hydration, decreased unpleasant sensory symptoms, and improved subjective skin conditions according to the subjects. Third, in all the studies, the product safety was very good. The tolerance was very good even in Asian subjects (data not shown), whose skin has been reported to be more chemo‐sensitive than other ethnic groups.^2^ Moreover, the product decreased IL‐1α induction by heat–cold stress at D0 and prevented S100A8/9 induction by heat–cold stress at D28. Although these two mediators have already been described as biomarkers in sensitive skin,^20^, ^21^ their high baseline level in our model triggers difficulties to show higher induction with the heat‐cold stress and thus also triggers difficulties to assess the product efficacy. In addition, in a RHE model, the active complex exhibited antioxidant properties and skin barrier protection effects by significantly preventing both the extra‐ and intra‐cellular oxidative stress induced (via MDA and H2DCF‐DA analysis, respectively) as well as the reduction in corneodesmosin level induced by urban dust and UVA exposure (Supplementary—Data S1).

Corneometry and TEWL are interesting parameters for evaluation of the skin barrier improvement after several days of a topical product application in SSS subjects.^6^, ^7^, ^22^ Contrary to hydration assessment, no significant variation was observed by TEWL analysis after 28 days of product application in 30 women (40% dry skin, 53% mixed, and 7% normal) compared with baseline and control conditions. However, in an in vivo study performed on the forearms of 22 women who all had very dry skin, the dermo‐cosmetic product significantly reduced the TEWL by 29% after 28 days of application compared with the untreated area (data not shown). The difference in results could be explained by the skin type of the subjects, with the product only having a beneficial impact in subjects with very dry skin potentially associated with skin barrier alteration. Moreover, in a RHE model, the corneodesmosin level remained unchanged despite UVA/urban dust stress demonstrating a skin barrier protection effect of the dermo‐cosmetic product, which also directly protected the epidermal nerve endings (Supplementary—Data S1).

Among the studies performed to date to evaluate topical products in SSS, few have assessed the safety,^23^, ^24^ while some have assessed the impact of physical factors such as cold,^12^ heat, friction,^13^ and after shaving,^11^ and the majority have evaluated chemical factors, such as lactic acid^6^, ^7^, ^9^ and capsaicin.^8^, ^10^, ^11^ Only one study, however, was performed versus a vehicle.^11^ Interestingly, our work includes two assessments involving physical stresses (stripping and heat–cold) and one with chemical stress (capsaicin) performed in comparison with an untreated control, which enables objective assessment of the product's efficacy in response to different types of triggering factors. This broad efficacy could be due to the properties of the active complex (*Salvia miltiorrhiza* flower/leaf/root extract, a tetrapeptide, carnosine, and tocopherol) exerting antioxidant properties and enhancing skin barrier function, as shown in the RHE model (Supplementary—Data S1). Nisbet et al. also evaluated two cosmetic products (cream with UV filters and serum) containing two anti‐inflammatory agents (panthenol and palmitamide MEA) and an epidermal differentiation agent (niacinamide) in a UVB‐irradiated RHE model.^12^ Contrary to our UVA/urban dust‐induced RHE model, IL‐8, TFNα, and PGE_2_ levels were increased. They also demonstrated that 1 h of pretreatment with the cream containing UV filters prevented the increase in cytokines, DNA damage, and apoptosis, probably due to the UV filters.

One of the components of the active complex of the investigated product, *Salvia miltiorrhiza* flower/leaf/root extract, significantly inhibits TRPV1 receptors (transient receptor potential vanilloid receptor 1) in sensory neurons (data not shown), and inflammation in various inflammation models (PGE2 induced by pollutant in keratinocytes, COX‐2 induced by pollutant in RHE, and redness induced by leukotriene B4 or cold stress in vivo) (data not shown). In addition, the tetrapeptide, a lipopeptide, increases in vitro epidermal differentiation proteins (involucrin, loricrin), and enzymatic activity (transglutaminase) (data not shown). Finally, two antioxidants are present, carnosine, a biomimetic dipeptide naturally present in organic tissues,^25^, ^26^ and tocopherol, one of the active forms of vitamin E and major endogenous antioxidant lipid in the skin, particularly present in the stratum corneum and sebum.^27^

Some topical products devised for SSS patients also contained TRPV1 inhibitors, such as 4‐t‐butylcyclohexanol and pimecrolimus.^8^, ^10^, ^11^, ^28^, ^29^ The diversity of triggering factors in SSS indeed suggests that there is an abnormal activation of sensory receptors of the transient receptor protein (TRP) family because these proteins are the only proteins known to be activated by physical and chemical factors.^4^, ^5^ For example, TRPV1 can be activated by both capsaicin and heat, while TRPM8 is activated by both cold and menthol.^5^ Moreover, one study demonstrated that a product containing a TRPV1 inhibitor induced a faster soothing effect than another product.^10^ Our studies confirm the potential of TRPV1 inhibitors in SSS for immediate effects and after 28 days of application, as demonstrated after minutes,^10^, ^11^ 1–11 days,^8^ as well as 4 weeks^29^ after the product application in other published studies. Inhibition of TRPV1 receptors contributes to targeting of the causes of SSS and consequently reduces the immediate symptoms and appears to decrease skin sensitivity after weeks of application.

Therefore, this dermo‐cosmetic product exerts a complementary action by acting on the two main causes of SSS, the neuroinflammation and the skin barrier. Considering the immediate relief of sensitive skin exposed to environmental factors, but also a better understanding of the causes for the prevention of long‐term symptoms, corresponds to an ecobiological approach. This approach is essential in dermatology since the skin is an ever‐evolving ecosystem in relation to its environment, subject to different types of factors. Consequently, this product contributes to improving the skin‐related quality of life of SSS patients by significantly reducing their BoSS score (data not shown).

The limitations of the study include the small sample sizes, only being performed on women, and the fact that sensitivity to one irritant does not necessarily predict sensitivity to others.^30^ Further controlled randomized studies are hence warranted to confirm the efficacy of this product. The results obtained are, however, promising and suggest a relevant degree of efficacy and very good safety of this dermo‐cosmetic product in these clinical settings. In conclusion, this study showed that topical application of a dermo‐cosmetic product could prevent and improve the skin state in SSS exposed to physical and chemical triggering factors.

## CONFLICT OF INTEREST STATEMENT

All authors are employees of NAOS Ecobiology Company (Aix‐en‐Provence, France).

## Supporting information


Data S1:

## Data Availability

The data that support the findings of this study are available from the corresponding author upon reasonable request.
