# Supplementary material for: Management of triggering factor effects in sensitive skin syndrome with a dermo‐cosmetic product
Source: J Cosmet Dermatol. 2024 Sep 18;23(12):4325–33. doi: 10.1111/jocd.16529 (PMC11626381; doi:10.1111/jocd.16529)
Supplement: Supplementary file 1 — Data S1: [file JOCD-23--s001.docx]

**Supplementary file S1**

**Materials & Methods**

***In vitro* UV-pollution pre-clinical study**

*Reconstituted human epidermis model*

Reconstituted human epidermis (RHE; QIMA Synelvia) were produced for eight days of air exposure of primary normal human keratinocytes obtained from an abdominal dermolipectomy of a healthy subject (provided by Genoskin). At D8, topical application of 0.6 % lactic acid (a known triggering factor of SSS) was performed to induce a barrier default and mimic sensitive skin, except for the control condition. Then, 4 hours later, topical applications of the vehicle alone or with the active complex present in the tested product (2 mg/cm²) were performed for the condition of interest. At D9, a new application of active complex or vehicle was applied before the stress. After 4 hours, the concerned RHEs were exposed to stress: urban dust (100 μg/mL; Sigma-Aldrich) and UVA exposure (9 J/cm²; 2 DEM) induced by a Bio-Sun device (Vilber Lourmat, Marne-la-Vallée, France). At D10, the RHE were harvested and analyzed to measure the malondialdehyde (MDA) level by GC/MS, and the dihydrodichlorofluorescein diacetate (H2DCF-DA) and corneodesmosin (CDSN1) levels by immunostaining.

*MDA level evaluation*

RHE were extracted by acid hydrolysis, and a PFB derivation was performed, followed by liquid/liquid extraction, with the organic phase dried under nitrogen at 60°C. The residues were resuspended in 50 µL of hexane. Separation by gas chromatography was achieved using a 30 m × 0.25 mm × 0.10 µm ZB-5HT capillary column, and helium was used as the carrier gas at a constant flow of 1 mL/min. Both the injector and transfer line temperatures were set to 250°C. Pulsed spitless mode (25 psi pulse) was used. After a 30 s hold at 50°C, the oven temperature was increased to 180°C at 25°C/min, then to 250°C at 5°C/min, followed by 300°C at 25°C/min, with a final hold for 10 min. Negative ion chemical ionization with methane reagent gas was performed using Selected Ion Monitoring (SIM).

*H2DCF-DA and corneodesmosin immunostaining*

RHE were fixed with a 4 % formaldehyde-containing buffer and were dehydrated in multiple baths with increasing concentrations of ethanol and then embedded in paraffin to generate. cross-sections with a microtome.

The sections were deparaffinized and incubated at 92°C, pH 6, in a retrieval target solution and cooled to room temperature in the same solution. After PBS-Tween-5 % milk saturation, the sections were incubated for 1 hour with the primary antibody (anti-corneodesmosin, Abcam). After several washes, the binding sites recognized by the primary antibody were revealed with a secondary fluorescent antibody (GAM-Alexa 488), and the cell nuclei were stained with DAPI solution. The sections were washed in PBS-Tween and mounted in an anti-fluorescence mounting medium.

RHE were introduced into plastic cryomolds to which drops of OCT had been added. The mold on top of the aluminum plate was placed on dry ice for rapid freezing. Cross-sections were generated with a microtome and a drop of ProLong™ medium was added to preserve the probe fluorescence signal.

Quantitative analyses were expressed as the means of triplicate data.

**Results**

To better understand the pollution impact on the physiological mechanism in terms of oxidative stress and skin barrier function, an *in vitro* RHE model was used with lactic acid to mimic sensitive skin and stressed with urban dust and UVA exposure for the pollution condition. The MDA quantity was significantly increased by the stress, and significantly decreased (by 56 %) in the presence of the active complex (48.3 vs. 34.6 ng/mg, respectively, of total proteins) (**Fig. S1A**). Similarly, the H_2_DCF-DA level was significantly increased by the stress, and significantly decreased by 75 % in the presence of the active complex (intensity ratio H_2_DCF-DA/DAPI: 1288 % vs. 397 %, respectively) (**Figs. S1B and S1D**), with a significant decrease with the vehicle compared with the stress condition (648 % vs. 1288 %). Concerning the skin barrier, the stress significantly reduced the level of corneodesmosin, which was partially restored only with the active complex, corresponding to a significant increase of 46 % compared with the stress alone condition (intensity ratio
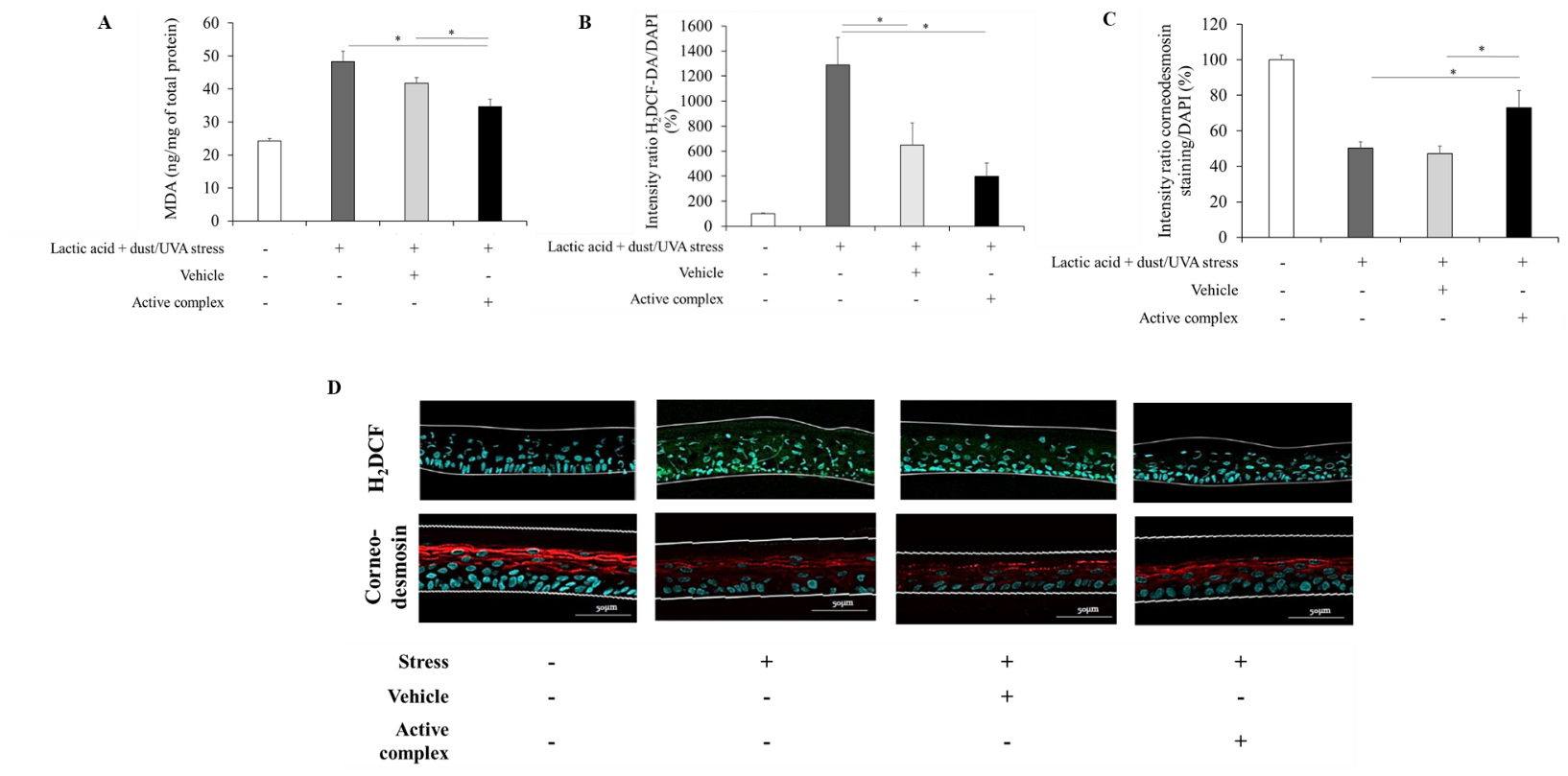
corneodesmosin/DAPI: 73.1 vs. 50.1 %, respectively) (**Figs. S1C and S1D**).

**Figure S1:** *In vitro* assessment of oxidative stress and skin barrier function of the active complex of the product under urban dust and UVA stress conditions via measurement of MDA (**A**), H2DCF-DA (**B**), and corneodesmosin (**C**) levels in an RHE model mimicking sensitive skin. Images of immunostaining of H2DCF-DA (green) and corneodesmosin (red) expression (blue: DAPI) (**D**). MDA, malondialdehyde; H2DCF-DA, dihydrodichlorofluorescein diacetate; RHE, reconstructed human epidermis. *p < 0.05
